# Study on the factors of hydrogen sulfide production from lignite bacterial sulfate reduction based on response surface method

**DOI:** 10.1038/s41598-023-47787-1

**Published:** 2023-11-23

**Authors:** Qigen Deng, Shuai Li, Mengmeng Yao, Chaosi Liu, Zhecheng Zhang, Sisi Xiang

**Affiliations:** 1 School of Safety Science and Engineering, Henan Polytechnic University, Jiaozuo, 454003 China; 2 State Key Laboratory Cultivation Base for Gas Geology and Gas Control, Henan Polytechnic University, Jiaozuo, 454003 China; 3 Collaborative Innovation Center of Coal Safety Production of Henan Province, Jiaozuo, 454003 China

**Keywords:** Microbiology, Environmental sciences

## Abstract

Bacterial sulfate reduction (BSR) is one of the key factors leading to the anomalous accumulation of hydrogen sulphide in coal mines. Environmental factors such as temperature and pH play a crucial role in the metabolism and degradation of coal by sulfate-reducing bacteria (SRB). In this study, coal samples were selected from Shengli Coal Mine, and SRB strains were isolated and purified from mine water using a dilution spread-plate anaerobic cultivation method. Based on single-factor experiments and response surface methodology (RSM), the impact of temperature, pH, oxidation–reduction potential (ORP), chemical oxygen demand to sulfate ratio (COD/SO_4_^2−^) on the generation of hydrogen sulphide during brown coal BSR was analyzed. The results showed that the anaerobic degradation of coal by SRB was inhibited by either too high or too low a temperature to produce hydrogen sulfide, and the greatest production of hydrogen sulfide occurred at a temperature of about 30 °C; The greatest production of hydrogen sulfide occurred at an initial ambient pH of 7.5; COD/SO_4_^2−^ ratio of around 2.0 is most conducive to hydrogen sulphide generation; the lower ORP value is more favorable for hydrogen sulfide generation. The optimal conditions obtained by RSM were: temperature of 30.37 °C, pH of 7.64 and COD/SO_4_^2−^ of 1.96. Under these conditions, the hydrogen sulfide concentration was 56.79 mg/L, the pH value was 8.40, the ORP value was −274 mV, and the SO_4_^2−^ utilization rate was 58.04%. The RSM results showed that temperature, ambient pH and COD/SO_4_^2−^ had a significant effect on hydrogen sulfide production, and the degree of effect was: ambient pH > temperature > COD/SO_4_^2−^.

## Introduction

The abnormal accumulation of hydrogen sulfide (H_2_S) in coal mines not only leads to sudden gas emissions, causing casualties, but also results in irreversible damage to the central nervous system of underground workers, leaving lasting effects. It can also have a significant impact on equipment, particularly in wet environments, where metal equipment is prone to 'hydrogen embrittlement' and similar issues^1,2^. BSR by sulfate-reducing bacteria (SRB) under anaerobic reduction conditions involves the absorption of sulphate, the oxidation of coal organic matter, and the transfer of electrons from organic substances to absorbed sulphates across the cell membrane. This process is considered one of the significant factors contributing to the formation of hydrogen sulphide in coal mines ^3^, drawing the attention of researchers.

Sulfate-reducing bacteria are highly sensitive to the environment, and environmental parameters such as environmental pH, temperature, COD/SO_4_^2−^, and ORP value have important effects on the metabolic activity of SRB for sulfate reduction^4^. Wang et al.^5^ showed that the SRB activity and the reduction efficiency of SO_4_^2−^ showed a trend of increasing and then decreasing with the increase of temperature. Tian et al.^6^ showed that the genus SRB has a wide range of temperature variation and many strains can grow metabolically at 10–40 °C, but there are also strains that can grow at 50–92 °C. Song et al.^7^ investigated the growth status of SRB at different temperatures and found that the best cell growth status of SRB was achieved at a temperature of 37 °C and the most hydrogen sulphide was produced in the media. Song et al.^7^ found that the best cell growth status of SRB and the most hydrogen sulfide production in the culture medium was observed at a temperature of 37 °C. Yan et al.^8^ isolated a sulfate-reducing strain SRB-1 from sludge, whose optimal growth temperature was determined to be 45 °C and optimal pH was determined to be 7.5. Sharma et al.^9^ found that sulfate-reducing bacteria had the highest sulfate reduction rate near neutral pH (6.5–7.5) and that SRB activity decreased at higher or lower pH values. Janyasuthiwong et al.^10^ showed that pH is the most important parameter affecting the lifetime and performance of sulfate reduction inverse fluidized bed reactors. Su et al.^11^ found that no SRB can be grown with O_2_ as an electron acceptor, and the ORP value for SRB growth must be below −100 mV. Chang et al.^12^ investigated the effect of ORP on bacterial community and corrosion rate and found that sulfide, corrosion current density and the proportion of SRBs in microbial community decreased with the increase of ORP, and O_2_ inhibited the growth of SRBs. The COD/SO_4_^2−^ value is an important indicator that affects the proportion of SRBs in the microbial community. Theoretically, the reduction of SO_4_^2−^ by SRB has a good effect at COD/SO_4_^2−^ = 0.67, and the actual COD/SO_4_^2−^ value is greater than 0.67 if the competition of other microorganisms for the electron community is considered^13^. Liu et al.^14^ found that the maximum removal of sulfate and heavy metals was achieved at COD/SO_4_^2−^ = 1.5. Liu et al.^14^ found that the removal rate of sulfate and heavy metals reached the maximum when COD/SO_4_^2−^ = 1.5.

In the current studies on SRB, the main focus has been on microbial management of wastewater ^15,16^, microbially influenced corrosion (MIC) of metals ^17,18^, and hydrogen sulfide generation in crude oil ^19,20^, with less research on hydrogen sulfide generation in coal mine BSR. Most previous studies have focused on the impact of individual factors, such as temperature and environmental pH, on the production of hydrogen sulphide by coal mine BSR. However, there is limited research on the interactions and the degree of influence among temperature, environmental pH, ORP value, and COD/SO_4_^2−^ in the actual process of hydrogen sulphide production by coal mine BSR. The study of the degree of influence of each factor on the process of hydrogen sulfide generation in BSR of coal mines and the interaction relationship between each factor plays an important role in the change pattern of hydrogen sulfide release in coal mines.

Therefore, based on single-factor and response surface methods, this study analyzed the effects of temperature, environmental pH, ORP values, and COD/SO_4_^2−^ on the formation of hydrogen sulfide in coal mine BSR. Simultaneously, by examining changes in hydrogen sulfide concentration, pH, ORP values, and sulfate removal rate, the study analyzed the degree of influence and interaction between various factors in the process of hydrogen sulfide production. This provides essential foundational support for supplementing and enhancing the understanding of the origin of coal mine hydrogen sulfide gas and the prevention of hydrogen sulfide disasters.

## Materials and methods

### Experimental materials

Strain enrichment: 10 mL of mine water sample was added to a prepared liquid culture medium, composed of 1 L of distilled water, potassium dihydrogen phosphate (0.5 g), ammonium chloride (1.0 g), sodium sulfate (1.0 g), calcium chloride (0.05 g), magnesium chloride hexahydrate (2.0 g), yeast extract (1.0 g), ascorbic acid (0.1 g), sodium thioglycolate (0.1 g), ferrous sulfate heptahydrate (0.5 g), and sodium lactate (1.1 g). Seal the liquid with liquid paraffin and place it in a constant temperature incubator for enrichment cultivation. Repeat the above process until the liquid culture medium turns black in color, emits a foul egg odor, and the lead acetate test paper turns black, indicating the successful enrichment of sulfate-reducing bacteria. Employ a dilution spread-plate anaerobic cultivation method to isolate and purify the enriched bacterial culture until obtaining sulfate-reducing bacterial strains.

Medium preparation process: the following chemical reagents were weighed using an electronic balance: potassium dihydrogen phosphate (0.5 g), ammonium chloride (1.0 g), sodium sulfate (1.0 g), calcium chloride (0.05 g), magnesium chloride hexahydrate (2.0 g), yeast extract (1.0 g), ascorbic acid (0.1 g), sodium thioglycolate (0.1 g), ferrous sulfate heptahydrate (0.5 g), and sodium lactate (1.1 g). These reagents were added to a beaker containing 1 L of distilled water and stirred until completely dissolved. The pH of the medium was adjusted to 7.0 ± 0.1 at 25 °C. The mixture was sterilized under high pressure at 121 °C for 15 min and deoxygenated by purging with nitrogen gas for 5 min. All of the chemical reagents mentioned above were purchased from Chengjiu Ke Laboratory.

Coal sample preparation: the coal samples were collected from Victory Coal Mine, which is located in the outskirts of Xilinhot, belonging to the northern part of the New Huanxiashan Formation in the Xilingol League. The geological structure in this area forms a monocline inclined in the northwestern direction with a gentle dip. The coal samples were crushed to a particle size between 60 and 80 mesh, sterilized with 75% alcohol for 15 min, and then dried at 80 °C for 24 h in a vacuum drying oven before sealing for storage. See Table 1 for coal analysis details.

**Table 1:** The basic properties of the coal (%).

Coal	Mad	Aad	Vad	FCad
Shengli Coal Mine	10.96	11.05	28.57	45.42

### Experimental methods

Effect of temperature on the formation of hydrogen sulfide in BSR: 10 g of lignite was put into a 250 mL conical flask with inactivated bacteria, 200 mL of bacterial suspension with 3% inoculum was configured in an anaerobic workstation, nitrogen was passed, the initial pH value of 7.0 ± 0.1 and the initial ORP value of −100 mV were adjusted, sealed, and the gas collection bag was connected. The incubators were incubated at 20, 25, 30, 35 and 40 ℃ for 20 days, and the collection bags were changed every 2 days and appropriate amount of liquid was taken to measure the concentration of hydrogen sulfide, pH, ORP and SO_4_^2−^ availability to analyze the effect of temperature on the formation of hydrogen sulfide in BSR.

Effect of environmental pH on BSR hydrogen sulfide formation: the initial pH in the conical flask was adjusted with 1 mol/L HCI and 1 mol/L NaOH, making the initial pH values 6.0, 6.5, 7.0, 7.5 and 8.0. The initial ORP value −100 mV was adjusted and incubated in a constant temperature incubator at 30 °C. The changes in parameters during the formation of hydrogen sulfide in BSR were measured every 2 days.

Effect of COD/SO_4_^2−^ on hydrogen sulfide formation in BSR: the initial COD/SO_4_^2−^ in the solution was adjusted by adjusting the SO_4_^2−^ content in the conical flask so that the initial COD/SO_4_^2−^ was 1.0, 1.5, 2.0, 2.5 and 3.0. The initial pH was adjusted to 7.0 ± 0.1 and the initial ORP value −100 mV and incubated in a constant temperature incubator at 30 °C. Parameters during the formation of hydrogen sulfide in BSR were measured every 2 days.

Effect of ORP value on hydrogen sulfide formation in BSR: the initial ORP values in conical flasks were adjusted to −100 mV, −150 mV, −200 mV, −250 mV and −300 mV with cysteine, and the initial pH value was adjusted to 7.0 and incubated in a constant temperature incubator at 30 ℃. Samples were taken every 2 days to determine the indicators of hydrogen sulfide formation process in BSR.

Based on the results of single-factor experiments, we conducted experiments using the Box–Behnken design (BBD). We selected temperature (A), initial pH (B), and COD/SO_4_^2−^ (C) as the response factors for response surface methodology (RSM), with total hydrogen sulphide concentration, pH value, ORP value, and SO_4_^2−^ utilization rate as the response variables. Table 2 summarizes these factors and their respective levels. Experimental parameters and results are presented in Table 3.

**Table 2:** Factors and levels used for Box–Behnken design.

Factors	Coded levels		
	− 1	0	+ 1
Temperature (℃)	25	30	35
pH value	7.0	7.5	8.0
COD/SO_4_^2−^	1.5	2.0	2.5

**Table 3:** Results of response surfaces.

No.	Variable			Response value			
	Temperature (A)	pH value (B)	COD/SO_4_^2−^ (C)	H_2_S (mg/L)	pH value	ORP value	Utilization of SO_4_^2−^ (%)
1	25	7.0	2.0	34.1	7.63	−230	39.2
2	35	7.0	2.0	36.8	7.70	−250	44.8
3	25	8.0	2.0	37.1	8.10	−261	44.5
4	35	8.0	2.0	35.9	8.20	−263	46.1
5	25	7.5	1.5	42.1	7.84	−253	45.7
6	35	7.5	1.5	44.9	7.94	−265	47.3
7	25	7.5	2.5	43.6	7.88	−256	47.6
8	35	7.5	2.5	46.4	7.98	−258	49.2
9	30	7.0	1.5	39.8	8.12	−234	42.3
10	30	8.0	1.5	42.6	8.62	−283	46.4
11	30	7.0	2.5	41.4	8.16	−251	45.2
12	30	8.0	2.5	44.1	8.68	−245	45.5
13	30	7.5	2.0	58.0	8.32	−270	58.5
14	30	7.5	2.0	57.6	8.32	−270	58.0
15	30	7.5	2.0	57.2	8.33	−272	59.1
16	30	7.5	2.0	57.0	8.32	−272	58.3
17	30	7.5	2.0	58.0	8.34	−274	57.9

### Parameter detection method

The concentration of hydrogen sulfide was measured by gas chromatography using Agilent 7890B ^21^; pH was measured by FE28 pH meter; ORP value was measured by ORP-422 type redox potential meter; SO_4_^2−^ is determined by barium chromate spectrophotometry (HJ/T 342−2007) ^22^.

## Results and discussion

### Effect of temperature on the formation of hydrogen sulfide in BSR

**Figure 1:**
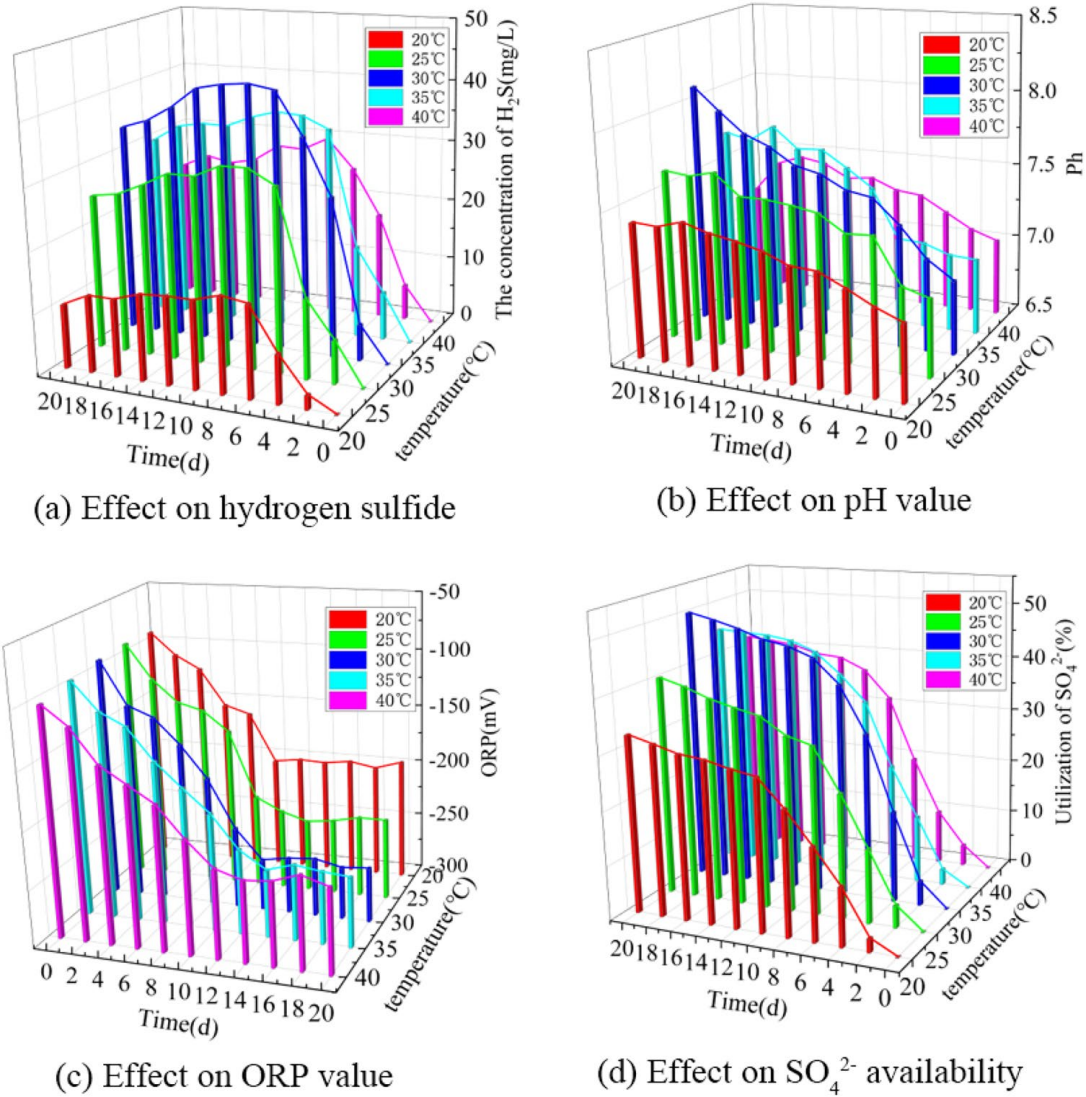
Effect of temperature on the formation of hydrogen sulfide in BSR. (**a**) Effect on hydrogen sulfide. (**b**) Effect on pH value. (**c**) Effect on ORP value. (**d**) Effect on SO_4_^2−^availability.

Based on the single-factor experimental data, a 3D bar chart of the effect of temperature on the formation of hydrogen sulfide in BSR was obtained, as shown in Fig. 1.

As can be seen from Fig. 1a, the concentration of hydrogen sulfide at different temperatures first increased and then slightly decreased as the experimental time was extended. At 0 to 2 days, the hydrogen sulfide concentration grew slowly; at 2 to 8 days, the hydrogen sulfide concentration grew rapidly to the maximum; at 8 to 12 days, the hydrogen sulfide concentration stabilized near the maximum; at 12 to 20 days, the concentration of hydrogen sulfide began to show a small decrease. When the experimental temperatures were 20, 25, 30, 35 and 40 °C, the maximum values of hydrogen sulfide concentration were 15.5, 32.1, 42.6, 36.1 and 29.8 mg/L, respectively, from which it can be seen that the concentration of hydrogen sulfide showed a trend of increasing and then decreasing with the rise of temperature from 20 to 40 °C, indicating that either too high or too low temperature would inhibit the anaerobic degradation of lignite by sulfate-reducing bacteria to produce hydrogen sulfide. When the temperature is too low, it leads to lipid waxing of the cell membrane, reducing the activity of membrane proteins and thus limiting the ability of the cell membrane to transport electron donors and electron acceptors^23^. In contrast, when the temperature is too high, microorganisms undergo heat shock reactions, which also cause blunting of protein enzymes and lead to the death of sulfate-reducing bacteria^24^. Therefore, it can be inferred that the sulfate-reducing bacteria used in this experiment are medium-temperature bacteria, and the optimum temperature for hydrogen sulfide production from lignite BSR is about 30 °C.

As can be seen from Fig. 1b, the pH increased to some extent at different temperatures as the experimental time increased. After 20 days of incubation at 20, 25, 30, 35 and 40 °C, the pH values increased to 7.40, 7.63, 8.10, 7.70 and 7.20, respectively. This is due to the reaction of sulfate ions with the desulfurization of sulfate-reducing bacteria to produce bicar bonate, which subsequently undergoes hydrolysis to produce hydroxide, thereby increasing the pH of the reaction system^25^. The most pronounced increase in pH was observed at 30 °C, indicating the highest SRB activity and the most vigorous metabolism.

As shown in Fig. 1c, the ORP values at different temperatures had roughly the same change pattern as the experimental time was extended, and all showed a trend of first decreasing and then stabilizing. After 20 days of incubation, the ORP values of the solutions were −195, −230, −253, −240 and −227 mV, respectively. at 30 °C, the ORP values decreased most obviously and the SRB metabolism was the most vigorous. Between 20 and 40 °C, there was a significant decrease in ORP values of the solutions, and all of them were less than −100 mV, indicating that SRB could survive between 20 and 40 °C.

As can be seen from Fig. 1d, the utilization rate of SO_4_^2−^ increased with the extension of the experimental time, and the slope of the change curves all showed a trend of increasing and then decreasing, indicating that the sulfate ion as an electron acceptor was reduced to produce hydrogen sulfide from slow to fast to slow. After 20 days of incubation, the utilization of SO_4_^2−^ reached 32.8, 40.5, 50.3, 45.2, and 41.7%, respectively. The utilization of SO_4_^2−^ was lower at 20 °C and 25 °C because the low temperature did not sufficiently stimulate the activity of enzymes and membrane proteins in the bacteria, thus affecting the efficiency of SRB in reducing sulfate to produce hydrogen sulfide^21^. At 30 °C, the utilization of SO_4_^2−^ by SRB reaches a maximum, and as the incubation temperature increases, the activity of the enzyme system and the ability to operate on electron donors and electron acceptors in SRB gradually increases, thus increasing the efficiency of SRB in metabolizing SO_4_^2−^ to produce hydrogen sulfide. At temperatures above 30 °C, the breakage of weak non-covalent bonds such as hydrogen bonds and van der Waals forces that partially maintain the active center and higher structure of the enzyme leads to a decrease in enzyme activity or even inactivation. Therefore, at 35 °C and 40 °C, the utilization of SO_4_^2−^ by SRB decreases instead. Tang ^26^ isolated SRB strain S-7 from acid mine wastewater, and its optimum temperature was 30 °C. The results of this study are consistent with the above findings.

In summary, the most hydrogen sulfide was formed in lignite BSR at a temperature of about 30 °C. At 30 °C, after 20 days of incubation, the highest concentration of hydrogen sulfide produced was 42.6 mg/L, the pH was 8.10, the ORP value was −253 mV, and the utilization of SO_4_^2−^ was 50.3%.

### Effect of environmental pH on the formation of hydrogen sulfide in BSR

**Figure 2:**
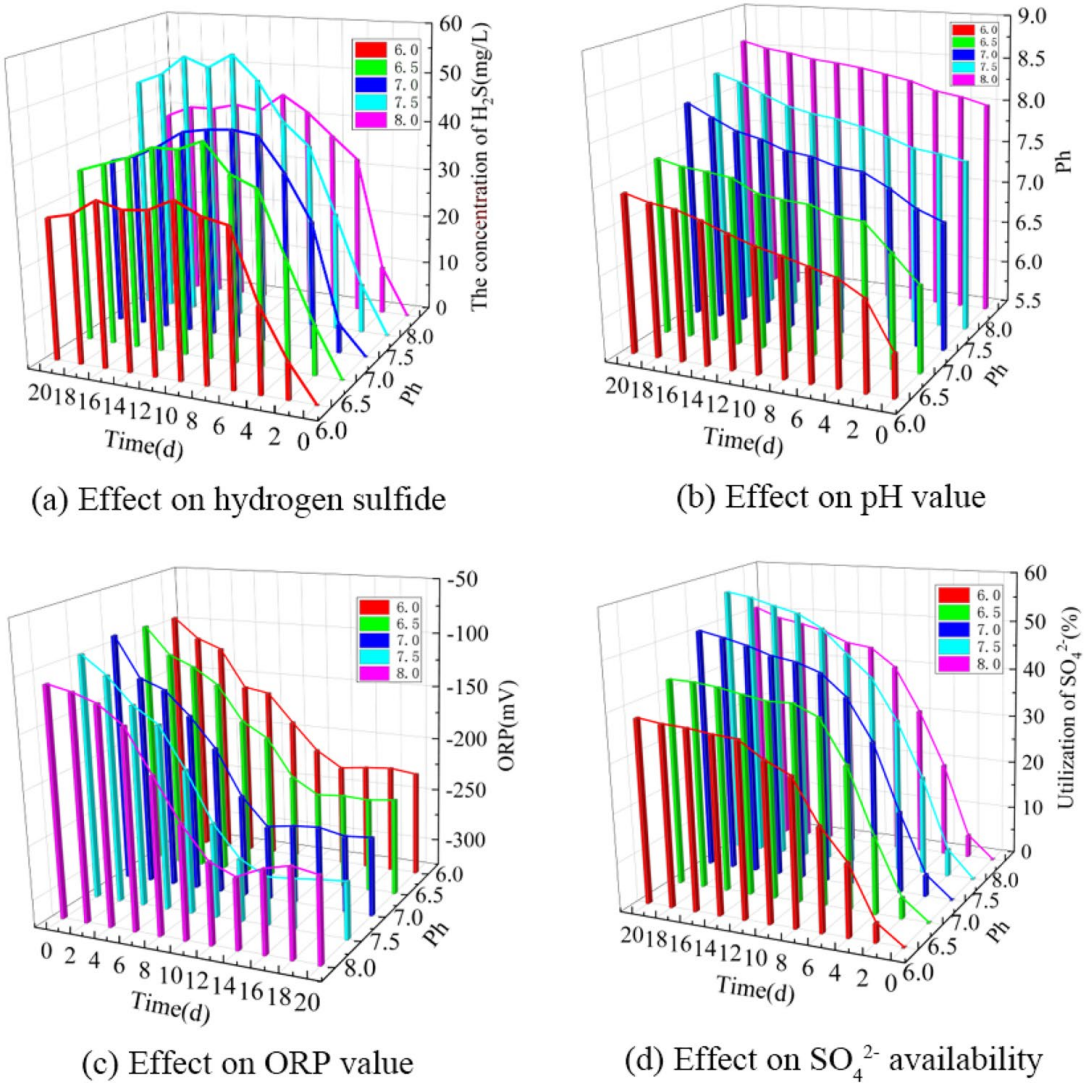
Effect of pH on the formation of hydrogen sulfide in BSR. (**a**) Effect on hydrogen sulfide. (**b**) Effect on pH value. (**c**) Effect on ORP value. (**d**) Effect on SO4^2−^ availability.

Based on the single-factor experimental data, a 3D bar chart of the effect of environmental pH on the formation of hydrogen sulfide in BSR was obtained as shown in Fig. 2.

As can be seen from Fig. 2a, the concentration of hydrogen sulfide at different initial ambient pH values first increased and then slightly decreased as the experimental time was extended. Ambient pH is one of the main factors that strongly affects the metabolic activity of microorganisms and has a large effect on the anaerobic degradation of lignite by SRB to produce hydrogen sulfide^27^. When the initial ambient pH was 6.0, 6.5, 7.0, 7.5 and 8.0, the maximum values of hydrogen sulfide concentration could reach 35.0, 42.0, 42.6, 55.0 and 44.8 mg/L, respectively, from which it can be seen that the hydrogen sulfide concentration showed a trend of increasing and then decreasing with the increase of the initial ambient pH. At the initial ambient pH of 6.0, the hydrogen sulfide concentration changed slowly, indicating that the acidic pH inhibits the anaerobic degradation of lignite by SRB to produce hydrogen sulfide. Lower ambient pH has been reported to affect the activity of enzyme systems and protein synthesis in SRB^28^. Lower ambient pH also results in excessive pH gradients in the extracellular and intracellular environments of SRB, which requires most of the energy from redox processes to maintain pH homeostasis, which is extremely detrimental to SRB growth and metabolism^29^. The concentration of hydrogen sulfide reached a maximum when the initial ambient pH was 7.5, indicating that neutral to alkaline conditions are more suitable for coal degradation. When the initial ambient pH was 8.0, the concentration of hydrogen sulfide showed another decrease, indicating that the overly alkaline environment was also unfavorable for the anaerobic degradation of coal-produced hydrogen sulfide by SRB. It is known that the optimum initial ambient pH for lignite BSR hydrogen sulfide generation is about 7.5.

As shown in Fig. 2b, when the initial ambient pH was 6.0, 6.5, 7.0, 7.5, and 8.0, the pH of the solution increased to 7.38, 7.60, 8.10, 8.32, and 8.60 after 20 days of incubation. the increase in solution pH was mainly related to the metabolism of SRB. Guo et al.^30^ found that the reduction of SRB in the SO_4_^2−^ process increases the pH in solution, which is generally consistent with the results of the present study.

As can be seen from Fig. 2c, when the initial ambient pH was 6.0, 6.5, 7.0, 7.5 and 8.0, the ORP values of the solutions decreased to −228, −236, −253, −273 and −247 mV after 20 incubations, respectively. when the ambient pH was 7.5, the ORP values decreased most significantly, indicating that the neutral-alkaline conditions were more suitable for SRB reproduction and metabolism.

As shown in Fig. 2d, the utilization of SO_4_^2−^ by SRB increased significantly with the increase of experimental time at different initial environmental pH values. When the initial ambient pH was 6.0, 6.5, 7.0, 7.5 and 8.0, the utilization of SO_4_^2−^ by SRB reached 37.9, 42.8, 50.3, 56.3 and 51.0% after 20 days of incubation, respectively, from which it can be seen that the initial ambient pH has a great influence on the reduction of SO_4_^2−^ by SRB. When the initial ambient pH was 6.0, the metabolic efficiency of SRB for SO_4_^2−^ was relatively slow, and less hydrogen sulfide was produced. When the initial ambient pH was 7.5, the SRB had the highest utilization efficiency of SO_4_^2−^, and the utilization efficiency was more than 50%. When the initial ambient pH was 8.0, the utilization of SO_4_^2−^ by SRB showed a decrease, and it can be seen that both acidic pH and alkaline pH inhibit the efficacy of SRB in reducing sulfate to produce hydrogen sulfide. Extreme pH has been reported to lead to disruption of cellular homeostasis and affect protein synthesis, while affecting the activity and stability of enzymes in the cell, thus affecting the progression of SO_4_^2−^ metabolism by SRB^31,32^.

Thus, the lignite BSR produced the most hydrogen sulfide at an initial ambient pH of about 7.5. At an initial ambient pH of 7.5, the highest concentration of hydrogen sulfide production was 55.0 mg/L after 20 days of incubation, with a pH of 8.32, an ORP of −273 mV, and a utilization rate of 56.3% for SO_4_^2−^.

### Effect of COD/SO_4_^2−^ on hydrogen sulfide formation in BSR

**Figure 3:**
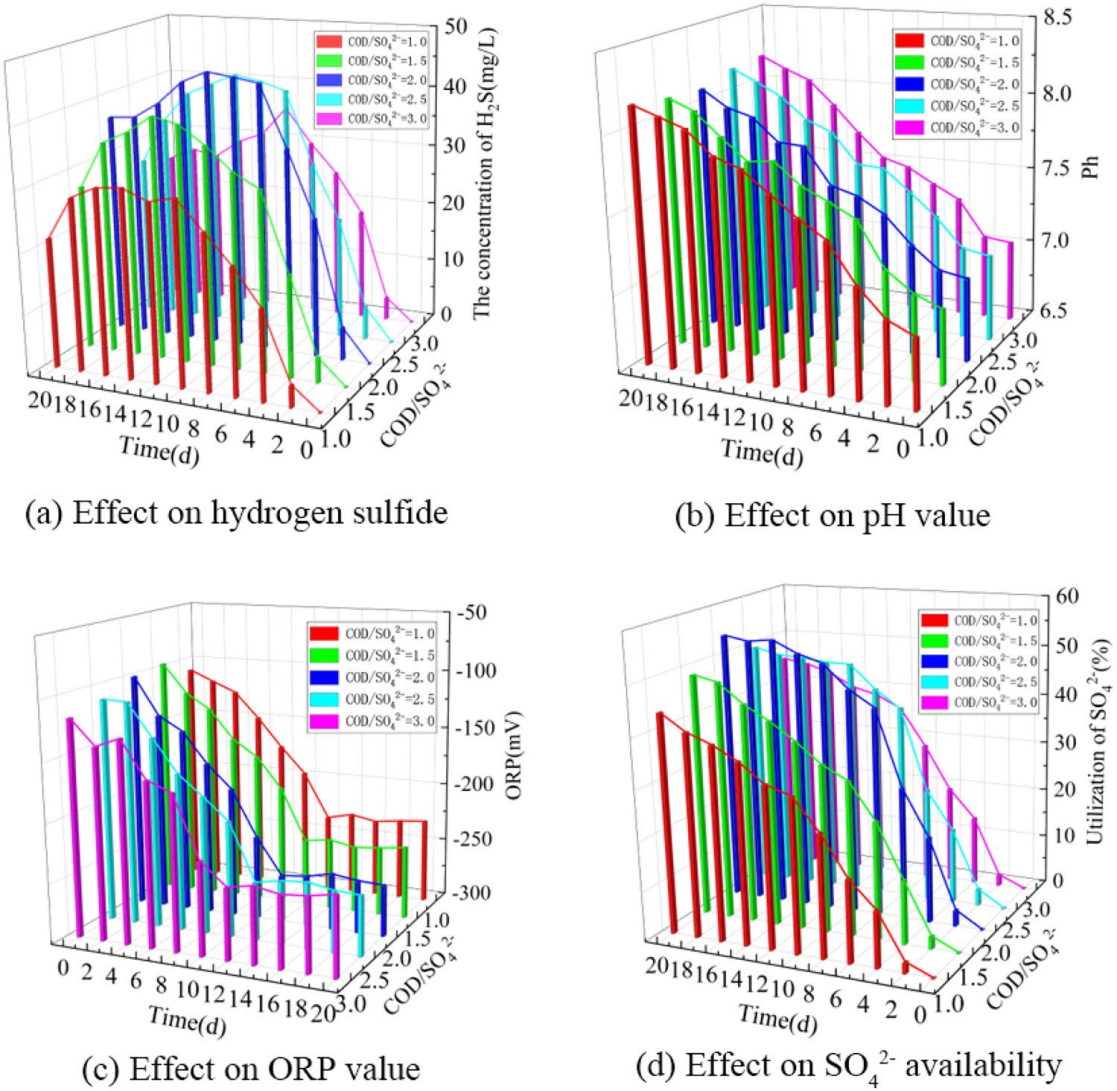
Effect of COD/SO_4_^2−^ on hydrogen sulfide formation in BSR. (**a**) Effect on hydrogen sulfide. (**b**) Effect on pH value. (**c**) Effect on ORP value. (**d**) Effect on SO_4_^2−^ availability.

Based on the single-factor experimental data, a 3D bar chart of the effect of initial COD/SO_4_^2−^ on hydrogen sulfide formation in BSR was obtained as shown in Fig. 3.

As can be seen from Fig. 3a, the hydrogen sulfide concentration first increased and then slightly decreased under different COD/SO_4_^2−^ conditions as the experimental time was extended. When the initial COD/SO_4_^2−^ was 1.0, 1.5, 2.0, 2.5 and 3.0, the maximum values of hydrogen sulfide concentrations reached 30.9, 39.8, 45.2, 42.9 and 35.6 mg/L. When the initial COD/SO_4_^2−^ was between 1 and 3, the hydrogen sulfide concentrations showed a trend of increasing and then decreasing with the rise of COD/SO_4_^2−^. When the initial COD/SO_4_^2−^ was 2.0, the most hydrogen sulfide was produced by SRB degradation of lignite. The results indicated that when the initial COD/SO_4_^2−^ was close to 2.0, which was most favorable for the growth and metabolism of SRB, the most hydrogen sulfide was produced by SRB degrading lignite. It was reported that 0.67 g COD is theoretically required to reduce 1 g SO_4_^2−^, but considering the competition between other bacteria and SRB for substrate during the actual culture, the required COD/SO_4_^2−^ ratio is much larger than the theoretical value^33^. The present experiments showed that SRB metabolism was most vigorous at an initial COD/SO_4_^2−^ of about 2.0, and the reduction of sulfate produced the most hydrogen sulfide. Dong et al. ^22^ showed that the SRB cell count was highest and metabolism was most vigorous at an initial COD/SO_4_^2−^ of 2.0.

As can be seen from Fig. 3b, the pH of the solution showed a gradual increase with the extension of the experimental time under different initial COD/SO_4_^2−^ conditions. When the initial COD/SO_4_^2−^ was 1.0, 1.5, 2.0, 2.5 and 3.0, respectively, the solution pH increased to 8.16, 8.12, 8.10, 8.16 and 8.18 after 20 days of incubation. thus, it can be seen that the change patterns of solution pH under different initial COD/SO_4_^2−^ conditions were not very different.

As can be seen from Fig. 3c, with the extension of the experimental time, the ORP values of different initial COD/SO_4_^2−^ have roughly the same change pattern all showing a trend of first decreasing and then stabilizing. After 20 days of incubation, the ORP values of the solutions were −235, −244, −260, −251 and −240 mV, respectively. when the initial COD/SO_4_^2−^ was 2.0, the ORP values decreased the most, indicating that the SRB metabolic activity was the most vigorous.

As seen in Fig. 3d, the utilization of SO_4_^2−^ by SRB increased significantly with the increase of experimental time at different initial COD/SO_4_^2−^. When the initial COD/SO_4_^2−^ values were 1.0, 1.5, 2.0, 2.5 and 3.0, respectively, the utilization of SO_4_^2−^ by SRB reached 43.9, 48.6, 54.2, 49.6 and 45.0% after 20 days of incubation. The utilization of SO_4_^2−^ by SRB showed a trend of increasing and then decreasing when the initial COD/SO_4_^2−^ was between 1.0 and 3.0, and the utilization of SO_4_^2−^ was above 40% with little difference, indicating that the initial COD/SO_4_^2−^ was not the main factor affecting the rate of SO_4_^2−^ reduction by SRB.

It is known that the most suitable initial COD/SO_4_^2−^ for H_2_S production from lignite BSR is about 2.0. At an initial COD/SO_4_^2−^ of 2.0, the highest concentration of hydrogen sulfide production was 45.2 mg/L, pH was 8.10, ORP value was −260 mV, and SO_4_^2−^ utilization was 54.2% after 20 days of incubation.

### Effect of initial ORP value on hydrogen sulfide formation in BSR

**Figure 4:**
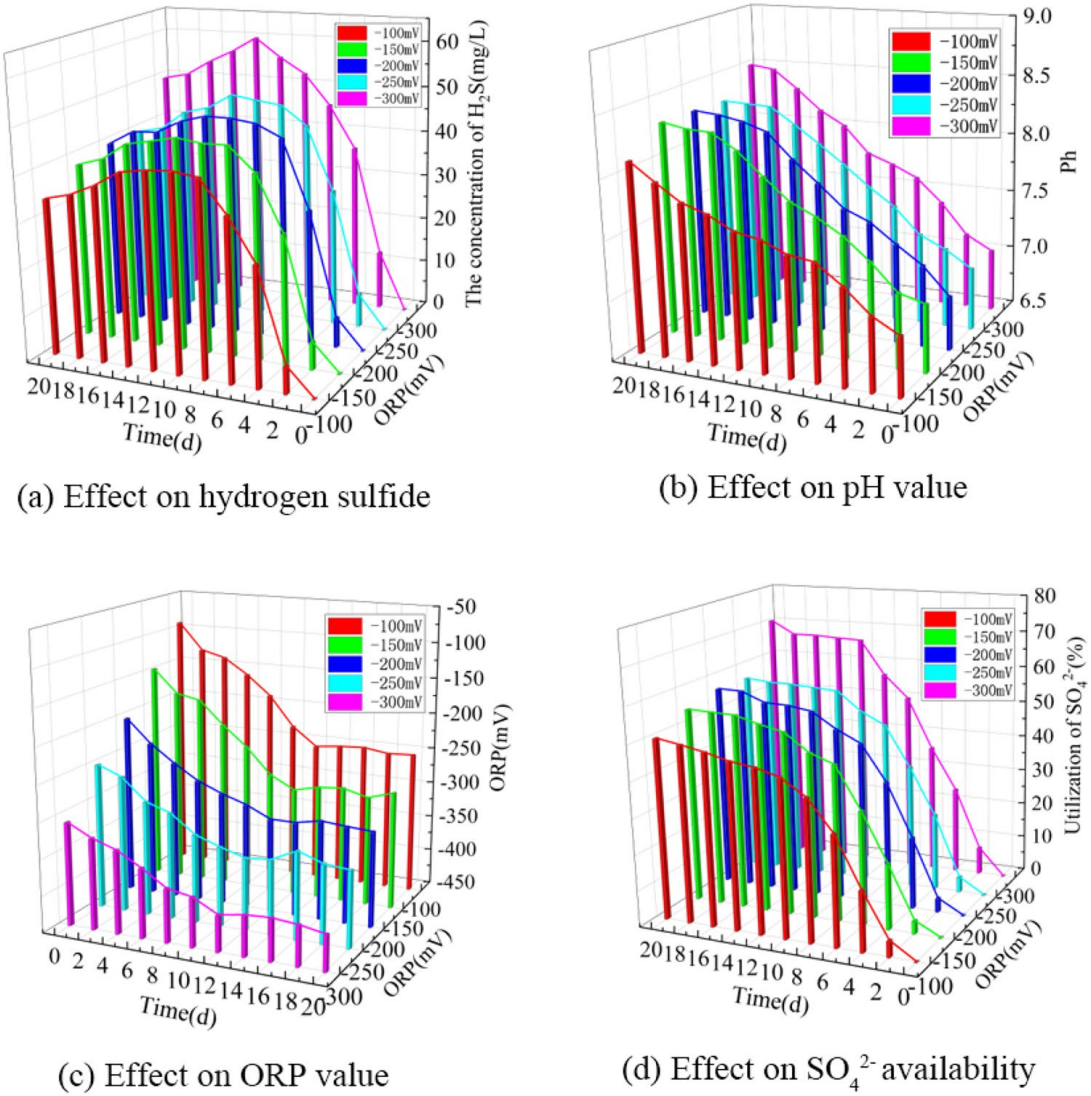
Effect of ORP on hydrogen sulfide formation in BSR. (**a**) Effect on hydrogen sulfide. (**b**) Effect on pH value. (**c**) Effect on ORP value. (**d**) Effect on SO_4_^2−^ availability.

Based on the single-factor experimental data, a 3D bar chart of the effect of the initial ORP value on the formation of hydrogen sulfide in BSR was obtained, as shown in Fig. 4.

As can be seen from Fig. 4a, the concentration of hydrogen sulfide increases and then decreases slightly at different initial ORP values as the experimental time is extended. When the initial ORP values were −100, −150, −200, −250 and −300 mV, the maximum values of hydrogen sulfide concentration could reach 42.6, 45.8, 47.5, 49.5 and 60.0 mg/L. When the initial ORP value was −100 mV, the concentration of hydrogen sulfide was larger, which indicated that SRB could carry out reproduction metabolism normally and reduce SO_4_^2−^ to produce hydrogen sulfide. As the ORP value decreased, the concentration of hydrogen sulfide became higher, indicating that the lower ORP value could promote the reproduction and metabolism of SRB and the production of hydrogen sulfide from lignite BSR. The hydrogen sulfide concentration was greatest when the initial ORP value was −300 mV, indicating that the SRB was the most active and metabolically efficient. Aoyagi et al. ^34^ showed that high ORP values decreased the relative abundance of SRB in the bioreactor. The present study is consistent with their results.

As can be seen from Fig. 4b, the pH of the solution increased continuously with the extension of the experimental time at different initial ORP values. When the initial ORP values were −100, −150, −200, −250 and −300 mV, the pH of the solution increased to 8.10, 8.30, 8.29, 8.27 and 8.50 after 20 days of incubation, respectively, and it can be seen that the pH of the solution increased more obviously when the initial ORP values were between −100 and −300 mV, indicating that SRB was able to carry out metabolic activities normally.

As can be seen from Fig. 4c, the ORP values under different initial ORP value conditions showed a trend of first decreasing and then stabilizing as the experimental time was extended. After 20 days of incubation, the ORP values of the solutions were −256, −300, −322, −351 and −401 mV, respectively. the ORP values of the solutions under different initial ORP value conditions all showed a significant decrease, indicating that the SRB had high activity.

As can be seen from Fig. 4d, the utilization of SO_4_^2−^ by SRB increased significantly with the increase of experimental time under different initial ORP values. When the initial ORP values were −100, −150, −200, −250 and −300 mV, after 20 days of incubation, the utilization of SO_4_^2−^ by SRB reached 50.3, 54.4, 56.6, 56.9 and 70.6%, respectively, from which it can be seen that when the ORP values were less than −100 mV, the SRB metabolism was more vigorous and the reduction of sulfate produced hydrogen sulfide was also higher.

In summary, when the initial ORP value was between −100 and −300 mV, the lower the initial ORP value was more favorable for the formation of hydrogen sulfide in lignite BSR. At an initial ORP value of −300 mV, the highest concentration of hydrogen sulfide produced was 60.0 mg/L after 20 days of incubation, with a pH value of 8.50, an ORP value of -401 mV, and a utilization rate of 70.6% for SO_4_^2−^.

### Response surface analysis of hydrogen sulfide formation in BSR

Multiple regression fitting of multiple factors affecting the formation of hydrogen sulfide in lignite BSR was carried out using Design expert 8.0 software, and the model was designed using the Box Behnken design (BBD) method. The temperature, ambient pH, and COD/SO_4_^2−^ were set as A, B, and C, respectively, and the response values of hydrogen sulfide concentration, pH, ORP value, and sulfate utilization rate under different conditions were used for multiple regression fitting, and the quadratic multinomial regression model was obtained as follows ^35^:

$$The \, concentrate  \, of  \, {H}_{2}S=57.56+0.89\times A+0.95\times B+0.76\times C-0.98\times A\times B+0.000\times A\times C-0.025\times B\times C-9.66\times {A}^{2}-11.93\times {B}^{2}-3.65\times {C}^{2},{R}^{2}=0.9963$$

$$pH \,  value=8.33+0.046\times A+0.25\times B+0.023\times C+0.0075\times A\times B+0.00\times A\times C+0.005\times B\times C-0.45\times {A}^{2}+0.033\times {B}^{2}+0.036\times {C}^{2},{R}^{2}=0.9994$$

$$ORP  \, value=-271.60-4.50\times A-10.88\times B+3.13\times C+4.50\times A\times B+2.50\times A\times C+13.75\times B\times C+7.93\times {A}^{2}+12.68\times {B}^{2}+5.67\times {C}^{2},{R}^{2}=9831$$

$$Utilization \,  of \,  {SO}_{4}^{2-}=58.36+1.30\times A+1.37\times B+0.73\times C-1.00\times A\times B+0.00\times A\times C-0.95\times B\times C-6.05\times {A}^{2}-8.65\times {B}^{2}-4.86\times {C}^{2},{R}^{2}=0.9942$$

**Figure 5:**
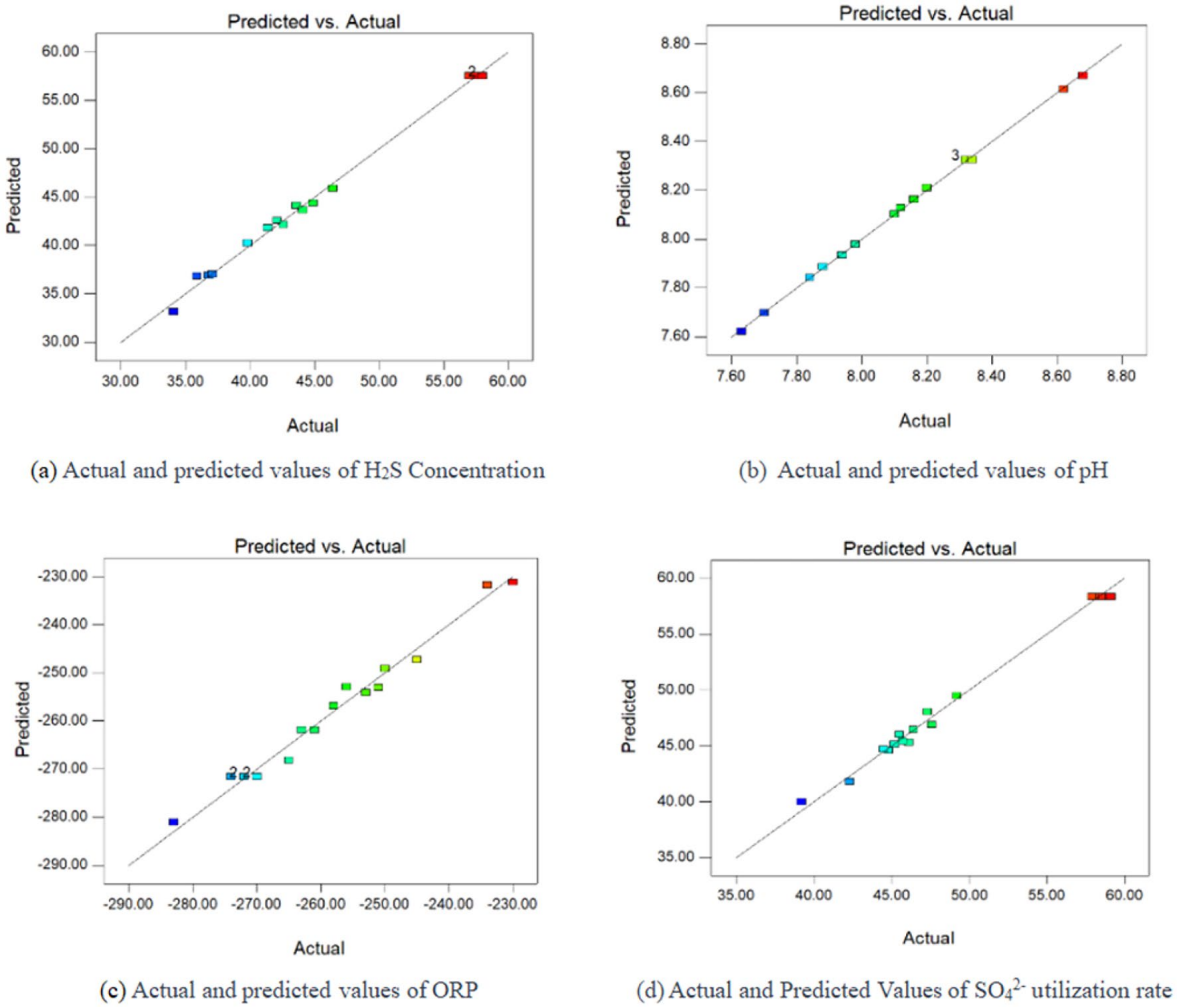
Actual and predicted values. (**a**) Actual and predicted values of H_2_S concentration. (**b**) Actual and predicted values of pH. (**c**) Actual and predicted values of ORP. (**d**) Actual and predicted values of SO_4_^2−^ utilization rate.

The results of the ANOVA and cumulative uncertainty are summarized in Table 4. The second-order model F values for hydrogen sulfide concentration, pH, ORP value and SO_4_^2−^ utilization under different experimental conditions were 207.34, 1346.99, 45.36 and 133.09, respectively, with P < 0.0001. This indicates that the above four second-order models fit well. The R^2^ values of the four models were 0.9963, 0.9994, 0.9831, and 0.9942, and the R^2^_Adj_ values were 0.9915, 0.9987, 0.9615, and 0.9867, respectively, indicating high model reliability and good fit. The misfit terms of the four models were 0.0632, 0.2593, 0.0711 and 0.0931, which were all greater than 0.05, and the models fit reasonably well. The coefficients of variation of the four models were 1.73, 0.13, 1.09 and 1.52%, which were all much less than 10%, indicating that the models had good credibility and accuracy^36^. Figure 5 further verifies that there is a very good match between the model predicted and actual values with a high degree of consistency, indicating that the model has a reliable and good fit. Therefore, the above four models can be used to analyze and predict the hydrogen sulfide concentration, pH value, ORP value and sulfate utilization under different conditions^37^.

**Table 4:** Results of the analysis of variance (ANOVA) for the response surface quadratic model.

Source	The concentration of H_2_S			pH			ORP			Utilization of SO_4_^2−^		
	F-value	p-value	Results	F-value	p-value	Results	F-value	p-value	Results	F-value	p-value	Results
Model	207.34	< 0.0001	Significant	1346.99	< 0.0001	Significant	45.36	< 0.0001	Significant	133.09	< 0.0001	Significant
A	10.07	0.0156	Significant	150.68	< 0.0001	Significant	20.45	0.0027	Significant	24.13	0.0017	Significant
B	11.54	0.0115	Significant	4358.60	< 0.0001	Significant	119.44	< 0.0001	Significant	27.00	0.0013	Significant
C	7.43	0.0295	Significant	35.66	0.0006	Significant	9.86	0.0164	Significant	7.51	0.0289	Significant
AB	6.08	0.0431	Significant	1.98	0.2021	Not significant	10.23	0.0151	Significant	7.14	0.0319	Significant
AC	0.000	1.0000	Not significant	0.000	1.0000	Not significant	3.16	0.1189	Not significant	0.00	1.0000	Not significant
BC	0.0025	0.9514	Not significant	0.88	0.3793	Not significant	95.47	< 0.0001	Significant	6.44	0.0388	Significant
A^2^	627.36	< 0.0001	Significant	7565.95	< 0.0001	Significant	33.38	0.0007	Significant	275.52	< 0.0001	Significant
B^2^	957.84	< 0.0001	Significant	40.99	0.0004	Significant	85.39	< 0.0001	Significant	562.94	< 0.0001	Significant
C^2^	89.91	< 0.0001	Significant	47.38	0.0002	Significant	17.12	0.0044	Significant	177.14	< 0.0001	Significant
Lack of Fit	5.69	0.0632	Not significant	1.98	0.2593	Not significant	5.27	0.0711	Not significant	4.40	0.0931	Not significant
R^2^	0.9963			0.9994			0.9831			0.9942		
R^2^_Adj_	0.9915			0.9987			0.9615			0.9867

**Figure 6:**
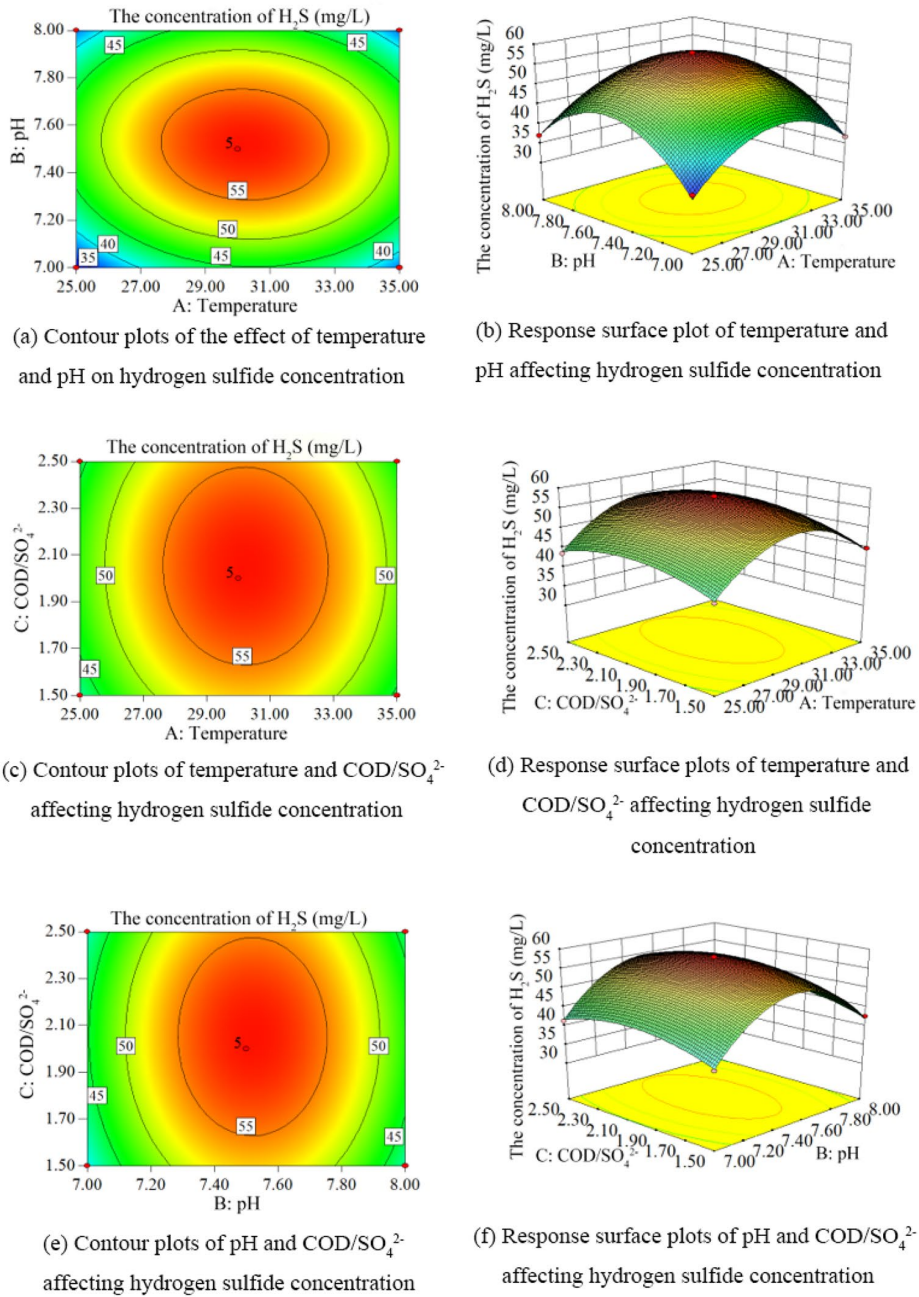
Contour and response surface plots of hydrogen sulfide concentrations under different conditions. (**a**) Contour plots of temperature and pH affecting hydrogen sulfide concentration (**b**) Response surface plots of temperature and pH affecting hydrogen sulfide concentration (**c**) Contour plots of temperature and COD/SO_4_^2−^ affecting hydrogen sulfide concentration. (**d**) Response surface plots of temperature and COD/SO_4_^2−^ affecting hydrogen sulfide concentration. (**e**) Contour plots of pH and COD/SO_4_^2−^ affecting hydrogen sulfide concentration. (**f**) pH and COD/SO_4_^2−^ influenced hydrogen sulfide concentration response surface plots.

According to the results of response surface experiments, the contours and response surface plots of hydrogen sulfide concentration under different conditions were obtained as shown in Fig. 6.

Figure 6 shows the combined effects of temperature (A), ambient pH (B) and COD/SO_4_^2−^ (C) on the formation of hydrogen sulfide in lignite BSR. From Fig. 6a, b, it can be seen that the hydrogen sulfide concentration shows a trend of increasing and then decreasing with the increase of temperature and ambient pH, and the contour plot is elliptical, which indicates a more obvious interaction between them. From Fig. 6c, d, it can be seen that the hydrogen sulfide concentration increases and then decreases with the increase of temperature and COD/SO_4_^2−^, and the variation of hydrogen sulfide concentration with temperature is steeper than that of COD/SO_4_^2−^, indicating that temperature has a greater influence on the formation process of hydrogen sulfide in lignite BSR. The contour lines are circular, which indicates that they have some interaction with each other. From Fig. 6e, f, it can be seen that with the increase of ambient pH and COD/SO_4_^2−^, the hydrogen sulfide concentration rises and then decreases, and the variation of hydrogen sulfide concentration with pH is steeper than that of COD/SO_4_^2−^, which indicates that the ambient pH has a greater influence on the formation process of hydrogen sulfide in lignite BSR. The contour lines were circular, indicating a certain interaction between them. The ANOVA showed that the single factors temperature, ambient pH and COD/SO_4_^2−^ had significant effects (P < 0.05) on the formation of hydrogen sulfide in lignite BSR, and the degree of influence was: ambient pH > temperature > COD/SO_4_^2−^. Its secondary term interaction AB had a significant interaction on hydrogen sulfide concentration (P < 0.05), and the degree of influence of the secondary term interaction on hydrogen sulfide concentration was: AB > BC > AC.

**Figure 7:**
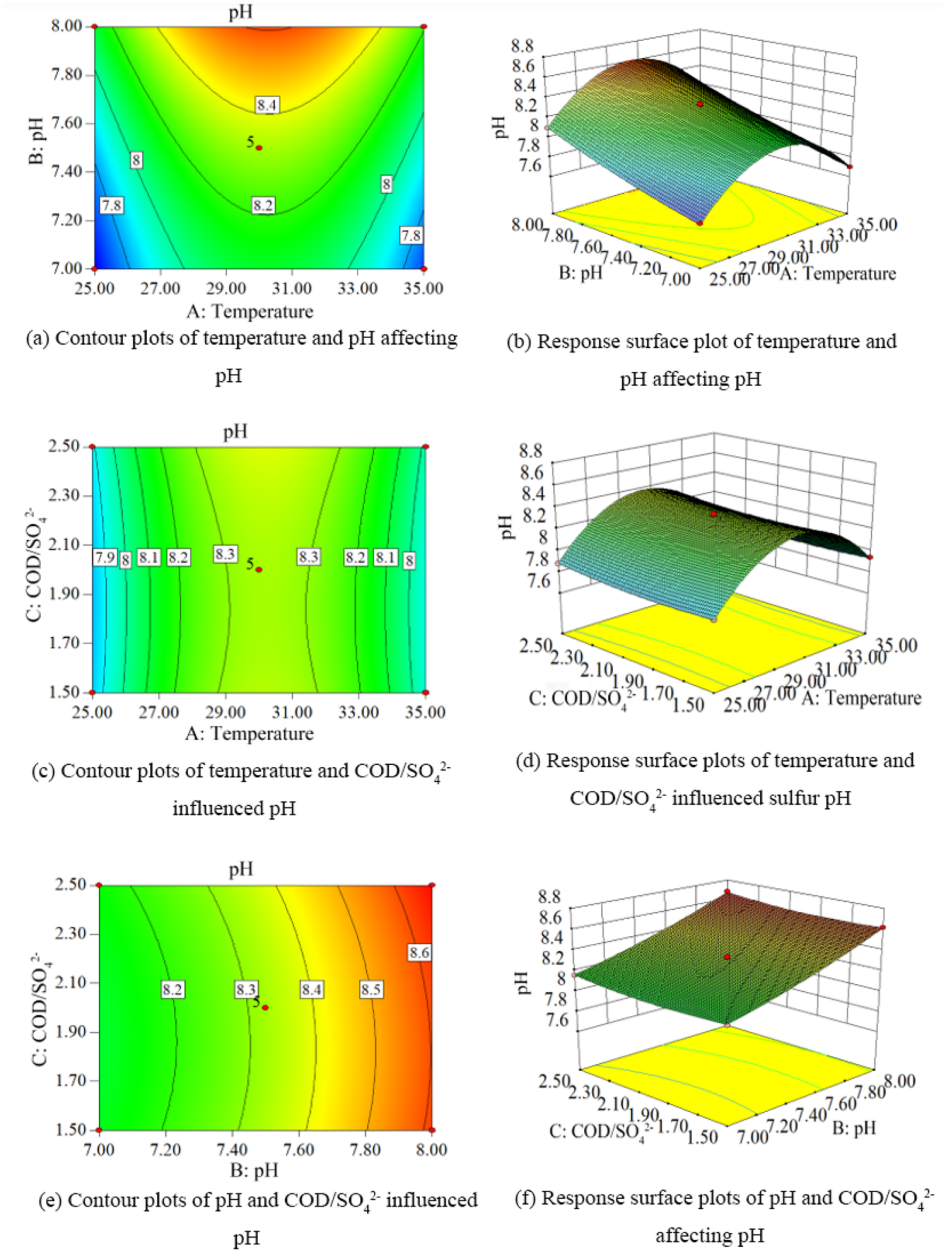
Contour and response surface plots of pH under different conditions. (**a**) Contour plot of temperature and pH affecting pH. (**b**) Response surface plot of temperature and pH affecting pH. (**c**) Contour plot of temperature and COD/SO_4_^2−^ affecting pH. (**d**) Response surface plot of temperature and COD/SO_4_^2−^ affecting sulfur pH. (**e**) Contour plot of pH and COD/SO_4_^2−^ affecting pH. (**f**) Contour plot of pH and COD/SO_4_^2−^ influenced pH response surface plots.

According to the results of response surface experiments, the contours and response surface plots of pH values under different conditions were obtained as shown in Fig. 7.

Figure 7 shows the combined effect of temperature (A), ambient pH (B) and COD/SO_4_^2−^ (C) on the solution pH during hydrogen sulfide formation in lignite BSR. When the temperature was 30.37 °C, the ambient pH value is 7.64 and COD/SO_4_^2−^ is 1.96, the final pH value of the solution is 8.40. The single factors temperature, ambient pH and COD/SO_4_^2−^ had highly significant effects (P < 0.001) on the solution pH during lignite BSR hydrogen sulfide formation, and the degree of effect was: ambient pH > temperature > COD/SO_4_^2−^. There was no interaction between temperature and ambient pH, temperature and COD/SO_4_^2−^, and ambient pH and COD/SO_4_^2−^ in response to pH during hydrogen sulfide formation in lignite BSR, with P values of 0.2021, 1.0000, and 0.3793, respectively.

**Figure 8:**
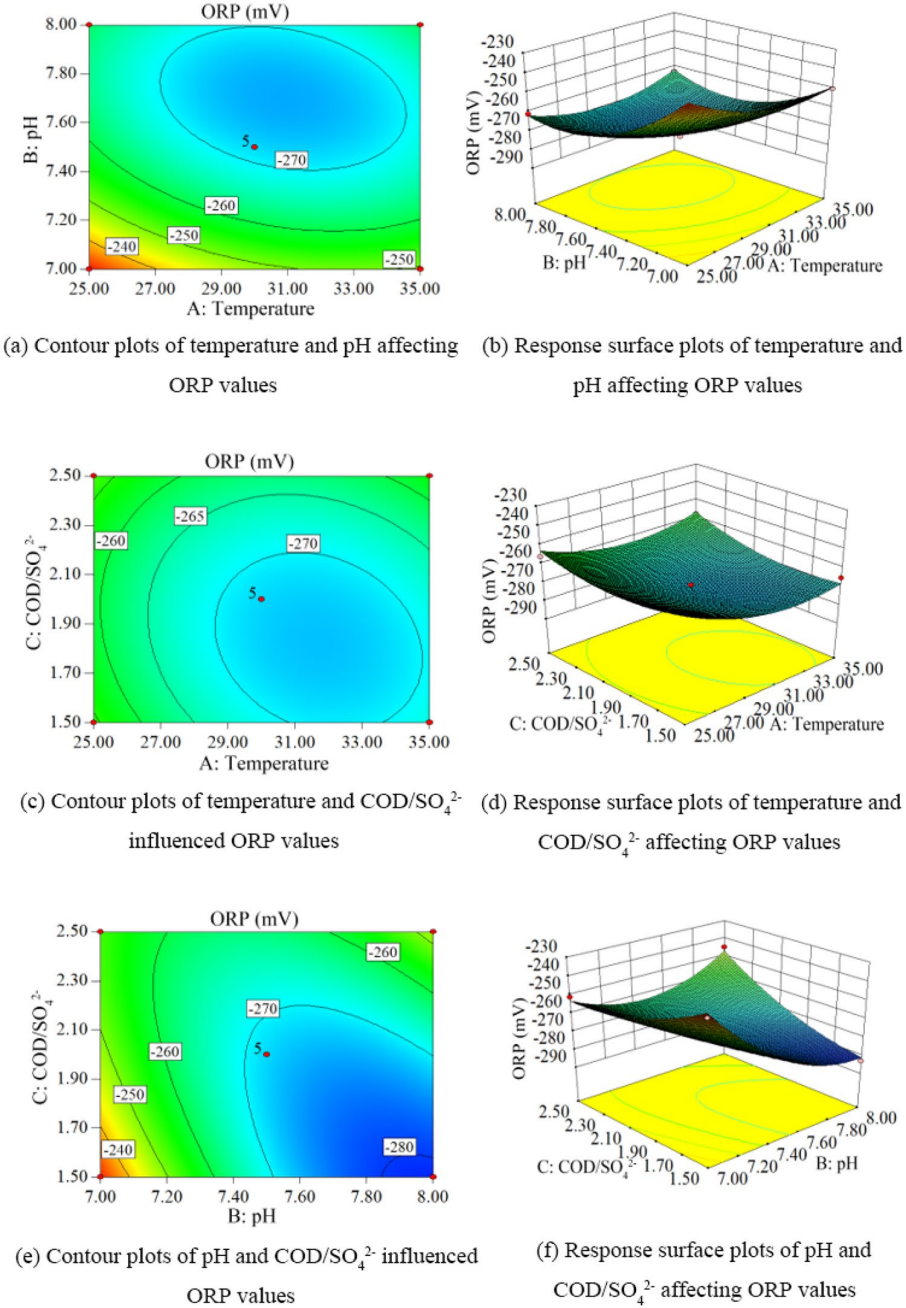
Contour and response surface plots of ORP values under different conditions. (**a**) Contour plot of temperature and pH affecting ORP values. (**b**) Response surface plot of temperature and pH affecting ORP values. (**c**) Contour plot of temperature and COD/SO_4_^2−^ affecting ORP values. (**d**) Response surface plot of temperature and COD/SO_4_^2−^ affecting ORP values. (**e**) Contour plot of pH and COD/SO_4_^2−^ affecting ORP values. (**f**) Contour plot of pH and COD/SO_4_^2−^ influenced ORP values response surface plots.

According to the results of response surface experiments, the contours and response surface plots of ORP values under different conditions were obtained as shown in Fig. 8.

Figure 8 shows the combined effect of temperature (A), ambient pH (B) and COD/SO_4_^2−^ (C) on the solution ORP values during hydrogen sulfide formation in lignite BSR. When the temperature was 30.37 °C, ambient pH was 7.64, and COD/SO_4_^2−^ was 1.96, the final ORP value of the solution was -274. The single factors temperature, ambient pH, and COD/SO_4_^2−^ had a significant effect (P < 0.05) on the ORP value of the solution during hydrogen sulfide formation in lignite BSR, and the degree of effect was: ambient pH > temperature > COD/ SO_4_^2−^. Among the effects on solution ORP values, there were significant interactions between temperature and ambient pH and ambient pH and COD/SO_4_^2−^, and the degree of influence of the secondary term interaction on hydrogen sulfide concentration was BC > AB > AC.

**Figure 9:**
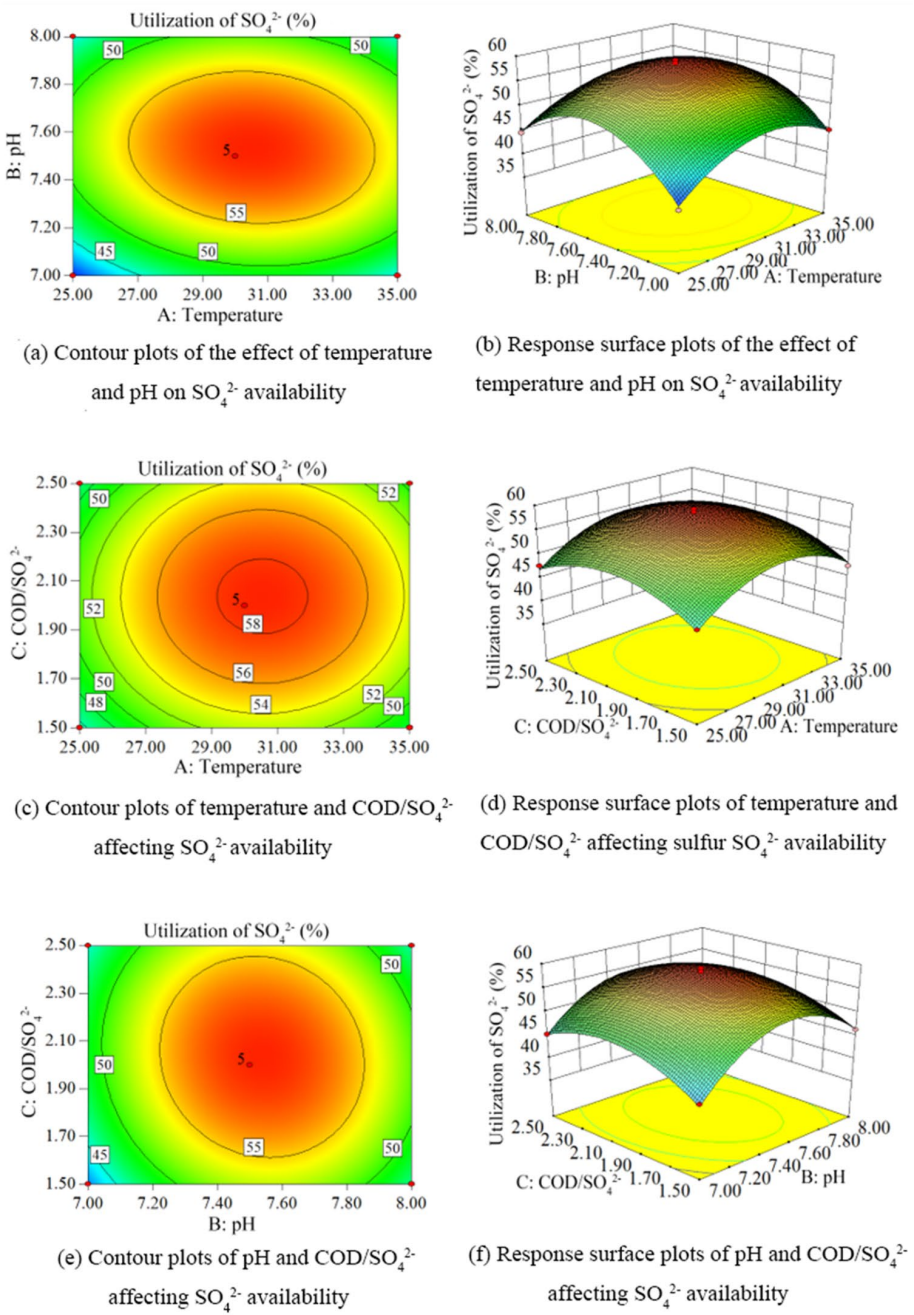
Contour and response surface plots of SO_4_^2−^ availability under different conditions. (**a**) Contour plot of temperature and pH affecting SO_4_^2−^ availability. (**b**) Response surface plot of temperature and pH affecting SO_4_^2−^ availability. (**c**) Contour plot of temperature and COD/SO_4_^2−^ affecting SO_4_^2−^ availability. (**d**) Response surface plot of temperature and COD/SO_4_^2−^ affecting sulfur SO_4_^2−^ availability. (**e**) Contour plot of pH and COD/SO_4_^2−^ affecting SO_4_^2−^ availability contour plots. (**f**) Response surface plots of pH and COD/SO_4_^2−^ affecting SO_4_^2−^ availability.

According to the results of response surface experiments, the contours and response surface plots of SO_4_^2−^ utilization under different conditions were obtained as shown in Fig. 9.

Figure 9 shows the combined effects of temperature (A), ambient pH (B) and COD/SO_4_^2−^ (C) on SO_4_^2−^ utilization during hydrogen sulfide formation in lignite BSR. From Fig. 9a, b, it can be seen that the SO_4_^2−^ utilization rate showed a trend of increasing and then decreasing with the increase of temperature and ambient pH, and the contour plot was elliptical, indicating a more obvious interaction between them. From Fig. 9c, d, it can be seen that SO_4_^2−^utilization increases first and then decreases with the increase of temperature and COD/SO_4_^2−^. The contour lines are circular, which indicates the interaction that they do not have with each other. From Fig. 9e, f, it can be seen that SO_4_^2−^use rises first and then decreases with the increase of ambient pH and COD/SO_4_^2−^, and the change of hydrogen sulfide concentration with pH is steeper than that of COD/SO_4_^2−^, indicating that the environmental pH has a greater influence on the formation process of hydrogen sulfide in lignite BSR. The contour lines were elliptical in shape, indicating a significant interaction between them. The ANOVA showed that the single factors temperature, ambient pH and COD/SO_4_^2−^ had significant effects (P < 0.05) on the formation of hydrogen sulfide in lignite BSR, and the degree of influence was: ambient pH > temperature > COD/SO_4_^2−^. Their secondary term interactions AB and BC had a significant interaction on hydrogen sulfide concentration (P < 0.05).

The optimum conditions were obtained by single-factor and response surface experiments: the temperature was 30.37 °C, the ambient pH was 7.64, and the COD/SO_4_^2−^ was 1.96. Under these conditions, the hydrogen sulfide concentration was 56.79 mg/L, the pH was 8.40, the ORP value was -274 mV, and the SO_4_^2−^ utilization rate was 58.04%. Considering the actual situation, the condition was modified to 30℃, the ambient pH value was 7.64, and the COD/SO_4_^2−^ was 1.96. After three parallel tests under the optimal condition again, the hydrogen sulfide concentration was 55.6 mg/L, pH value was 8.37, ORP value was -280 mV, and SO_4_^2−^utilization rate was 57.9%. The difference between the experimental and theoretical values is small, indicating that the model is good.

## Conclusion

1. In the process of brown coal BSR hydrogen sulfide formation, when the temperature is in the range of 20 to 40 °C, the concentration of hydrogen sulfide shows an initial increase followed by a decrease as the temperature rises. The maximum hydrogen sulfide production occurs at around 30 °C. With initial environmental pH values ranging from 6.0 to 8.0, hydrogen sulfide concentration initially increases and then decreases as the initial pH value rises, with the highest hydrogen sulfide production observed at an environmental pH of around 7.5. An initial COD/SO_4_^2−^ value of around 2.0 is most conducive to the degradation of brown coal by SRB to generate hydrogen sulfide. When the initial ORP value is less than -100 mV, SRB can carry out normal growth and metabolism. As the ORP value decreases, the hydrogen sulfide concentration increases.
2. Response surface analysis showed that temperature, ambient pH and COD/SO_4_^2−^ had significant effects on lignite BSR hydrogen sulfide formation, and the degree of effect was: ambient pH > temperature > COD/SO_4_^2−^. In the process of lignite BSR hydrogen sulfide formation, there was a significant interaction between temperature and ambient pH, and the interaction between temperature and COD/SO_4_^2−^ and ambient pH and COD/SO_4_^2−^ had no significant effect on lignite BSR hydrogen sulfide formation. The optimum conditions for the formation of hydrogen sulfide in lignite BSR were: temperature of 30.37 °C, ambient pH of 7.64, and COD/SO_4_^2−^ of 1.96. Under these conditions, the hydrogen sulfide concentration was 56.79 mg/L, pH was 8.40, ORP was −274 mV, and SO_4_^2−^ utilization was 58.04%.
3. The optimization of conditions for brown coal BSR hydrogen sulfide formation plays a crucial role in supplementing and enhancing our understanding of the existing origins of hydrogen sulfide gas in coal mines. It holds a certain degree of reference value for the prevention of hydrogen sulfide disasters, the improvement of environmental aspects in coal mining, and the protection of coal mine workers from the threats posed by hydrogen sulfide.

## Data Availability

The datasets used and analysed during the current study available from the corresponding author on reasonable request.
